# Quasi two-dimensional astigmatic solitons in soft chiral metastructures

**DOI:** 10.1038/srep22923

**Published:** 2016-03-15

**Authors:** Urszula A. Laudyn, Paweł S. Jung, Mirosław A. Karpierz, Gaetano Assanto

**Affiliations:** 1 Warsaw University of Technology, Faculty of Physics, Koszykowa 75, PL-00662 Warsaw, Poland; 2 NooEL–Nonlinear Optics and OptoElectronics Lab, University “Roma Tre”, I-00146 Rome, Italy; 3 Optics Lab, Physics Department, Tampere University of Technology, FI-33101 Tampere, Finland

## Abstract

We investigate a non-homogeneous layered structure encompassing dual spatial dispersion: continuous diffraction in one transverse dimension and discrete diffraction in the orthogonal one. Such dual diffraction can be balanced out by one and the same nonlinear response, giving rise to light self-confinement into astigmatic spatial solitons: self-focusing can compensate for the spreading of a bell-shaped beam, leading to quasi-2D solitary wavepackets which result from 1D transverse self-localization combined with a discrete soliton. We demonstrate such intensity-dependent beam trapping in chiral soft matter, exhibiting one-dimensional discrete diffraction along the helical axis and one-dimensional continuous diffraction in the orthogonal plane. In nematic liquid crystals with suitable birefringence and chiral arrangement, the reorientational nonlinearity is shown to support bell-shaped solitary waves with simple astigmatism dependent on the medium birefringence as well as on the dual diffraction of the input wavepacket. The observations are in agreement with a nonlinear nonlocal model for the all-optical response.

Optical solitons can result when the nonlinear response of a material counteracts diffraction and/or dispersion, leading to lightwave localization in space or time, respectively^1^,^2^,^3^. In homogeneous Kerr media with a pointwise intensity-dependent index of refraction which increases with the square of the electric field, solitons are stable in one transverse dimension (1D), e.g. in fibers or in planar waveguides. In media encompassing a periodic modulation of the refractive index in one or two transverse dimensions, waveguide arrays can support light propagation in the form of evanescently-coupled eigenmodes, with diffraction assuming a discrete character and self-confinement giving rise to discrete solitons. Due to a reduced effective dimensionality, discrete solitons can be observed at lower excitations than their continuous counterparts^4^,^5^. Discrete solitons have been demonstrated in 1D waveguide arrays and in two-dimensional (2D) triangular and hexagonal waveguide lattices^5^,^6^,^7^,^8^,^9^,^10^,^11^,^12^,^13^,^14^,^15^.

In this Paper, we consider a novel -propagation invariant- optical configuration where continuous diffraction in one transverse dimension and discrete diffraction in the orthogonal direction act together and yield the anisotropic spreading of an input (singly humped) optical wavepacket. In analogy with light localization in photo-inducted lattices^16^, particularly parabolic Weber-lattice solitons^17^, such “dual diffraction” due to different spreading mechanisms can be compensated by a self-focusing nonlinearity, eventually leading to astigmatic light localization across both transverse coordinates^18^. We study hereby dual diffraction and light localization in two transverse dimensions towards a novel class of self-localized, optical solitary waves in engineerable nonlocal media where the spreading of a finite light beam is no longer determined exclusively by its transverse size, but also by the periodic index modulation. Such localization is analogous to spatio-temporal confinement of ultrashort light pulses through the interplay of diffraction, dispersion and nonlinearity in periodic lattices^19^,^20^,^21^,^22^,^23^.

To achieve a “dual” diffractive behavior we employ chiral nematic liquid crystals with a periodic helical arrangement across *x* and uniform distribution across *y* and *z*, yielding discrete diffraction versus *x* and continuous diffraction versus *y*. The reorientational response of this molecular medium entails self-focusing and the formation of spatial solitary waves across *y*, as well as discrete localization across *x*. When the power levels required for both types of localizations are comparable, the structure supports the generation of quasi 2D spatial solitons with a finite degree of astigmatism linked to the two unequal diffractive processes and nonlinear responses.

## Geometry and Material

We consider the paraxial propagation of an optical wavepacket in the forward *z*-direction; the refractive index is taken to be periodic across *x* and nonlinear with the field, i.e. 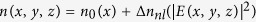 with 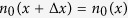 and ∆*n*_*nl*_ the intensity dependent contribution yielding self-focusing (or defocusing). Generally, in the linear regime one-dimensional Bloch waves (Floquet-Bloch waves) need be employed in the *x*-direction for a rigorous analysis of wave propagation in *yz*. However, using a more intuitive approach and aiming at the description of a realistic (weakly guiding) sample, the 1D periodic structure can be treated as an array of identical planar (graded-index) waveguides such that 
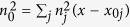
 and each j-th guide supports a single eigenmode *E*_*j*_ = Ψ_*j*_(*x* − *x*_0*j*_)exp(−*iβ*_*j*_*z*), with *β*_*j*_ = *β*, ∀*j*. Then the total electric field is the superposition 
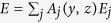
 with amplitudes *A*_*j*_ evolving versus propagation due to weak coupling between neighboring waveguides across *x* while undergoing continuous diffraction in *yz*.

The diffraction of a monochromatic wavepacket across *y* is determined by the initial spatial frequency spectrum (i.e. its waist), whereas the (discrete) diffraction across *x* is governed by period and amplitude of the refractive index modulation. An intensity-dependent index increase is responsible for self-focusing in both transverse directions. Therefore, in a *z* and *y*-invariant planar structure with refractive index modulation along *x*, nonlinear light localization into spatial solitons requires the proper engineering of both index modulation (period and contrast) and input beam width in order to compensate diffraction in *xz* and in *yz*, respectively. Nevertheless, such planar system is expected to yield stable 2D spatial soliton even in the limit of purely local Kerr nonlinearities^18^.

In order to realize the dielectric configuration described above, we used a thick layer of molecular soft matter, namely chiral nematic liquid crystals (ChNLC), exploiting their giant molecular nonlinearity, extended spectral transparency, high nonlocality, birefringence and adjustable refractive index periodicity in one dimension. Nematic liquid crystals consist of elongated molecules with a pronounced difference between the electronic polarizabilities when subject to electric fields along or orthogonal to the major molecular axes. In the nematic phase, with the molecules statistically aligned along a unity vector named *director* (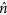) corresponding to the optic axis of the macroscopic uniaxial, the dielectric medium exhibits optical birefringence and, in the presence of intense fields, a large nonlinear response^30^. The latter stems from the Coulombian torque Γ which tends to align the induced dipoles (parallel to the director 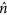) to the forcing field **E** until equilibrium is reached with the elastic intermolecular links characteristic of the liquid state: 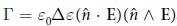, where 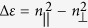 and *n*_||_ and *n*_⊥_ the refractive indices associated with electric fields along and perpendicular to 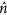, respectively. For light beams linearly polarized in the principal plane (defined by the propagation wavevector ***k*** and the optic axis 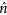), this *reorientational* response is large and self-focusing, able to support the formation of spatial solitons at mW or even sub-mW excitations^24^,^25^,^26^.

When a chiral dopant is added to nematic liquid crystals in a thin (a few tens of micrometers) planar cell with proper boundary conditions at the interfaces, the constituent organic molecules self-organize in anisotropic layers, with director rotated by a small (orientation) angle in successive layers (along *x*) and thereby forming a helix. Depending on anchoring conditions at the boundaries and material constants, these helical structures have a given pitch *p*, with *p* the distance along *x* after which the director 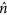 has completed a 2*π* rotation in the plane *yz*. ChNLC have been successfully employed to observe spatial solitons and their interactions^27^,^28^ as well as self-driven optical mode transformation^29^.

**Figure 1:**
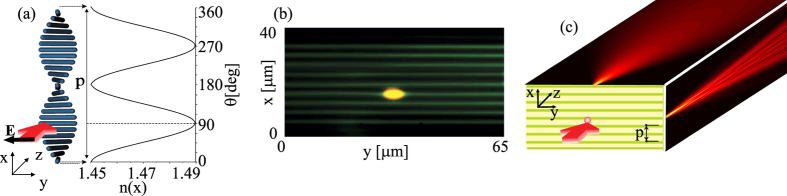
(**a**) 3D schematic of one pitch helical structure of the chiral nematic liquid crystal in a planar cell, with an incident light beam (red arrow) polarized along y and the periodic refractive index profile for an extraordinary wave. The blue rods represent the molecular director. (**b**) Photograph (through cross-polarizers) of the input section of the chiral sample (40 *μm*-thick ChNLC 1110) with a bell-shaped elliptical beam excitation at 793 *nm*; (**c**) Sketch of the ChNLC cell and its excitation, illustrating dual diffraction.

The sample geometry we consider here and used in the experiments is sketched in Fig. 1: in a 40*μ*m thick planar glass cell with planar anchoring at the inner interfaces, chiral-doped nematic liquid crystals form helices along *x*. A *y*-polarized light beam propagating along *z* experiences a refractive index continuously varying across the cell thickness *x* between its ordinary (*n*_⊥_) and extraordinary values (*n*_||_). Such graded-index structure is periodic with Δ*x* = *p*/2 and corresponds to a set of identical graded—index planar waveguides. Defining the orientation angle *θ* as the angle between the molecular director and the *y*-axis, 
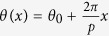
, the periodic distribution of the refractive index for extraordinary (*e*-) polarized light is given by


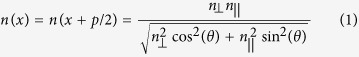


such *e*-index profile, constant in *yz* and graded along *x*, is plotted in Fig. 1(a) for the positive uniaxial case *n*_⊥_ = 1.45 and *n*_|| _= 1.49, corresponding to one of the chiral NLC materials employed in the experiments. Figure 1(b) is a polarization microscope photograph of the transverse (*xy*) cross-section of a ChNLC sample consisting of ten graded-index layers. A typical bell-shaped elliptical input beam is also visible in the photograph, launched with input wavevector along *z* and polarization along *y*, as illustrated in Fig. 1(c). When injected in *x* = *p*/2*π*(*νπ* *− θ*_0_) such that *n*(*x*) = *n*_||_, a beam of suitable wavelength experiences continuous diffraction in *yz* and discrete diffraction in *xz*. In the nonlinear regime, the *e-*polarized electric field can reduce the orientation angle *θ* and increase the refractive index, yielding self-focusing and, eventually, beam localization in *yz* (balancing out continuous diffraction^26^) and in *xz* (halting discrete diffraction by mismatching the input planar waveguide from the neighboring ones^5^). Hence, dual diffraction and self-focusing can give rise to anisotropic beam self-localization with the generation of astigmatic solitary waves.

## Results

**Figure 2:**
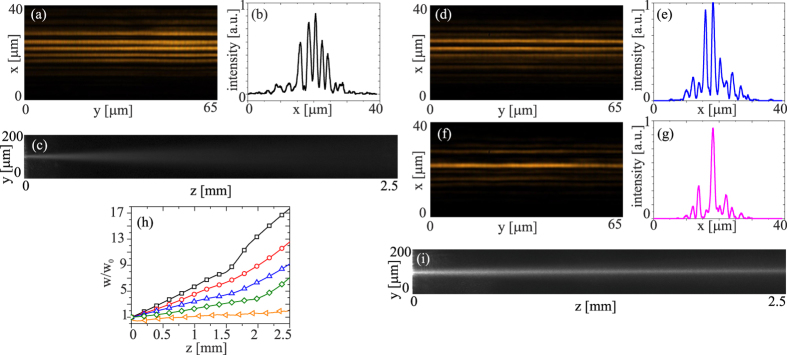
Experimental results for an input beam of waist *w*_0*x*_ = *w*_0*y*_ = 2.5 *μm* launched into one waveguide of the ChNLC 1110 structure. (**a**) *xy* output profile in *z* = 3.0 *mm* for power P < 1 mW; (**b**) corresponding cross-section across *x*; (**c**) beam evolution in *yz* for P < 1 mW; (**d**) *xy* output profile and (**e**) corresponding cross-section across *x* for P = 5 mW; (**f**) *xy* output profile and (**g**) corresponding cross-section across *x* for P = 30 mW; (**h**) measured (normalized) beam waist versus z for various excitations: black (squares) 1 mW, red (circles) 10 mW, blue (triangles) 20 mW, green (diamonds) 30 mW, orange (tilted triangles) 50 mW; (**i**) beam evolution in *yz* for P = 50 mW.

In the experiments we used a linearly-polarized Gaussian (*TEM*_00_) beam at *λ* = 793 *nm*. The beam was gently focused at the input of the planar sample using a microscope objective. In a first set of experiments we used low-birefringence chiral nematic liquid crystals, specifically the mixture 1110 with Δ*ε* = 0.1176 > 0 at room temperature; the addition of a chiral dopant resulted in a chiral arrangement with helical axes along *x* and pitch *p* = 8*μm*. Figure 2 illustrates beam propagation in the structure: an input beam with waist *w*_0*x*_ = *w*_0*y*_ = 2.5 *μm* is launched into a single layer and undergoes continuous diffraction in *yz* and discrete diffraction in *xz*, distributing its energy among various planar waveguides, Fig. 2(a); at the output a cross section of its profile along *x* (in *y* = 0, *z* = 3 *mm*) shows several peaks corresponding to the excited eigenmodes, Fig. 2(b). Owing to continuous diffraction in *yz*, the initial beam diverges and progressively reduces its intensity becoming hardly visible after a few Rayleigh ranges, Fig. 2(c). As the beam power increases and progressively mismatches the input waveguide, the number of guided modes carrying energy decreases across *x*, as visible in the output images Fig. 2(d,f) and in the corresponding intensity cross-sections along *x* in *z* = 3 *mm*, Fig. 2(e,g). At sufficiently high powers (>30 mW) light induced reorientation fully counteracts discrete diffraction via the induced mismatch, yielding self-localization across *x* and a discrete soliton (Fig. 2(g))^9^. Moreover, continuous diffraction/divergence in *yz* also reduces, with the ratio *w*/*w*_0_ going from ≈17 for *P* = 1 *mW* to ≈6 for *P* = 30 mW in *z* = 2.5 *mm*. Essentially, in the excitation interval [1, 30] mW the input beam gets localized in the *x* coordinate via discrete trapping, but only partially localized in the *y* dimension through continuous self-focusing. A further increase in input power, up to 50 mW, eventually yielded a spatial soliton in the plane *yz*, Fig. 2(i), but altered discrete propagation and the discrete soliton in *xz* due to a thermally assisted deformation of the structured medium (as detailed in the Supplementary Information section).

**Figure 3:**
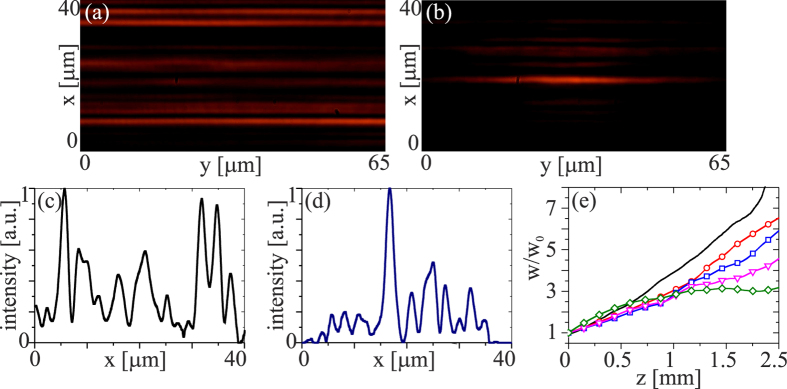
Experimental results in ChNLC 1110 for an elliptical input beam with waists *w*_0*x*_ = 3 *μm* and *w*_0*y*_ = 4 *μm*. (**a**) *xy* output profile at *z* = 3 *mm* for powers (**a**) <1 mW; (**b**) 20 mW; (**c**,**d**) corresponding intensity cross-sections along *x*; (**e**) measured (normalized) beam waist versus *z* for increasing excitations: black (solid line) 1 mW, red (circles) 10 mW, blue (squares) 15 mW; magenta (triangles) 20 mW, green (diamonds) 30 mW.

Having gathered evidence of partial self-localization in the transverse plane, but not a clear demonstration of a solitary wave in both *x* and *y* due to dual diffraction in the 1D-discrete/1D-continuous chiral metastructure, we resorted to elliptical beam excitation in order to reduce the mismatch in the power levels required to yield self-localization both in *x* and in *y*. To this extent we expanded the beam along *y* to lower beam divergence and the required power to obtain self-localization into a spatial soliton. Figure 3 illustrates the experimental results for a single-humped beam with *w*_0*y*_ = 4 *μm* and *w*_0*x*_ = 3 *μm* along the two transverse coordinates, respectively. Similar to the case presented above, at low power the beam experienced continuous diffraction in *yz* and discrete diffraction in *xz*. In the nonlinear regime, all-optical reorientation resulted both in a reduced number of excited waveguides (Fig. 3(a–d)) and in a progressively lower divergence along *y* (Fig. 3(e)). At sufficiently high excitations, the nonlinear response overcame dual diffraction and supported self-localization with light propagation in a single (input) waveguide of the array in *xz* and a non-diffracting spatial soliton across *y*. Figure 3(a,b) displays photographs of the output beam in the plane *xy* at low and high powers, respectively, illustrating the transition from discrete diffraction to a discrete soliton in the array; Fig. 3(c,d) shows the corresponding intensity profiles across *x*. As visible in the graphs of Fig. 3(e), the larger input waist across *y* (i.e. the lower input divergence) allowed obtaining an astigmatic spatial soliton at powers higher than 20 mW, with *w* ≈3*w*_0_ for *P* = 30 mW. In summary, after input beam tailoring, nonlinear reorientation mediated not only the gradual transition from discrete diffraction to localization, but also the emergence of a self-trapped beam in the orthogonal coordinate, where continuous diffraction was balanced out by self-focusing.

**Figure 4:**
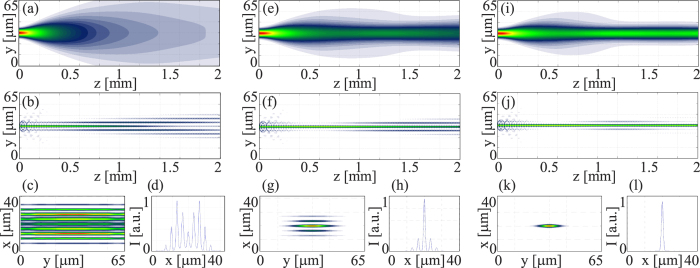
Numerical results for an elliptical beam with *w*_0*x*_ = 3 *μm* and *w*_0*y*_ = 4 *μm* in *x* and *y*, respectively, launched in chiral 1110. Normalized excitations 
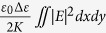
 are: (**a**–**d**) 1.2; (**e**–**h**) 4.9; (**i**–**l**) 45.7; (**a**,**e**,**i**) beam evolution in *yz*; (**b**,**f**,**j**) beam evolution in *xz*; (**c**,**g**,**k**) transverse output profiles in *z* = 2 *mm*; (**d**,**h**,**l**) corresponding profile cross-sections across *x*. Here the normalized power scales to mW excitation through a factor ≈60.

The observed generation of quasi-2D astigmatic solitons in ChNLC can be described by paraxial propagation in a non-homogeneous dielectric with the refractive index distribution Eq. (1) and the orientation angle 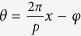 (we set *θ*_0_ = 0), with *φ* the light-induced (reorientational) perturbation:


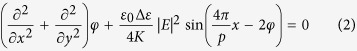


neglecting longitudinal field components^26^,^29^. Here *K* is the Frank strength in the single elastic constant approximation and rules the nonlocal response of the medium^30^,^31^. In our configuration and director orientation, the twist deformation is dominant as 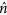 lays and remains in the plane *yz*^28^; hence, in Eq. (2) *K* = *K*_22_ (see Methods for numerical values). We resorted to the Beam Propagation Method with actual optical and geometric parameters of the sample, launching a single-hump Gaussian beam at 793 *nm* in the mid-plane (*x*: *θ* = *νπ*) of one of the graded-index waveguides. Figure 4 shows the numerical results for an elliptical beam with *w*_*ox*_ = 3 *μm* and *w*_*oy*_ = 4 *μm* and various powers. To calculate the evolution of the wavepacket envelope *E* we employed a finite difference algorithm with a 4th-order Runge-Kutta in propagation, using a relaxation method and a multigrid approach to solve Eq. (2).

In the linear limit, diffraction (continuous and discrete) is retrieved in both planes *yz* and *xz* (Fig. 4(a–d)]. As the excitation increases, the beam narrows across *y* due to self-focusing while the coupling length (strength of guide-to-guide tunneling) lowers in the array across *x*, progressively isolating the input waveguide from the neighboring ones (Fig. 4(e–h)). Eventually, the nonlinear beam self-traps across *y* forming a 1D spatial soliton (a *nematicon*) and across *x* forming a discrete soliton (a *discrete nematicon*), Fig. 4(i–l). The resulting self-confined wavepacket is a quasi-2D solitary wave with astigmatism dependent on the different amounts of spatial dispersion afforded by dual diffraction in the structure.

These numerical results are in excellent agreement with the experimental data and the predictions, although excitation values tend to differ with the experiments as in-coupling losses, scattering losses, thermal effects and alignment imperfections in the material were neglected in the model. Moreover, the role of the chiral dopant on the elastic constants and optical properties (hence the nonlinearity) of the ChNLC is unknown.

**Figure 5:**
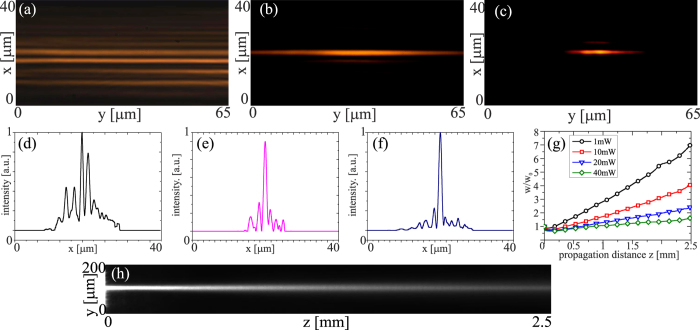
Experimental results in ChNLC 903 for a *y*-polarized elliptical beam with input waists *w*_0*x*_ = 3 *μm* and *w*_0*y*_ = 4 *μm* along *x* and *y*, respectively, launched in the core of a planar waveguide defined by the chiral structure. *xy* output image in *z* = 3 *mm* for powers (**a**) P < 1 mW; (**b**) 20 mW; (**c**) 40 mW; (**d**–**f**) corresponding intensity cross-sections along *x*; (**g**) measured beam waist *w*_*y*_ versus z for various excitations: black (circles) <1 mW; red (squares) 10 mW; blue (triangles) 20 mW; green (diamonds) 40 mW; (**h**) beam evolution in *yz* for P = 20 mW.

**Figure 6:**
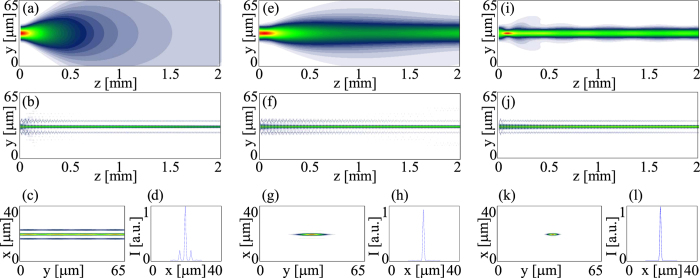
Numerical results for an elliptical beam with *w*_0*x*_ = 3 *μm* and *w*_0*y*_ = 4 *μm* in *x* and *y*, respectively, launched in the chiral NLC micture 903. Normalized excitations (defined as in Fig. 4) are (**a**–**d**) 0.01; (**e**–**h**) 1.5; (**i**–**l**) 3.1; (**a**,**e**,**i**) beam evolution in *yz*; (**b**,**f**,**j**) beam evolution in *xz*; (**c**,**g**,**k**) transverse output profiles in *z* = 2 *mm*; (**d**,**h**,**l**) corresponding profile cross-sections along *x*. Here the normalized power scales to mW excitation through a factor ≈27.

Another possibility towards quasi-2D solitons in the chiral structure relies on using soft matter with larger optical birefringence, such as to tune the coupling strength between waveguides in the array. A higher birefringence of the ChNLC, in fact, yields a larger contrast of the refractive index modulation defining the graded-index planar waveguides, i.e. a better confinement of the guided eigenmode(s) and a longer coupling distance (for the same pitch). To this extent, we employed the NLC mixture 903 with *n*_⊥_ = 1.47 and *n*_||_ = 1.55 (i. e. a birefringence *n*_||_ − *n*_⊥_ = 0.08 twice larger than in 1110) with a chiral dopant resulting in a pitch *p* = 8 *μm* as in the previous analysis. We launched an elliptical beam as in the experiments described above, with input waist *w*_0*x*_ = 3 *μm* and *w*_0*y*_ = 4 *μm* and monitored the beam evolution versus power. As anticipated, in the linear regime the light beam experienced discrete diffraction across *x* but with a smaller diffraction cone (i.e. smaller zero diffraction angle) due to the higher birefringence (see Fig. 5(a,d)). Power increments caused both progressive light decoupling from adjacent waveguides in the array across *x* and reduced beam divergence along *y* (Fig. 5(b,e,g,h)). For sufficiently high excitations of the order of 40 mW the beam underwent self-localization along both *y* and *x*, forming a quasi-2D soliton with lower astigmatism (Fig. 5(c,f,g)). Numerical simulations analogous to those shown in Fig. 4 were carried out with reference to the ChNLC 903 and are presented in Fig. 6. The overall trend is remarkably consistent with the measurements.

**Figure 7:**
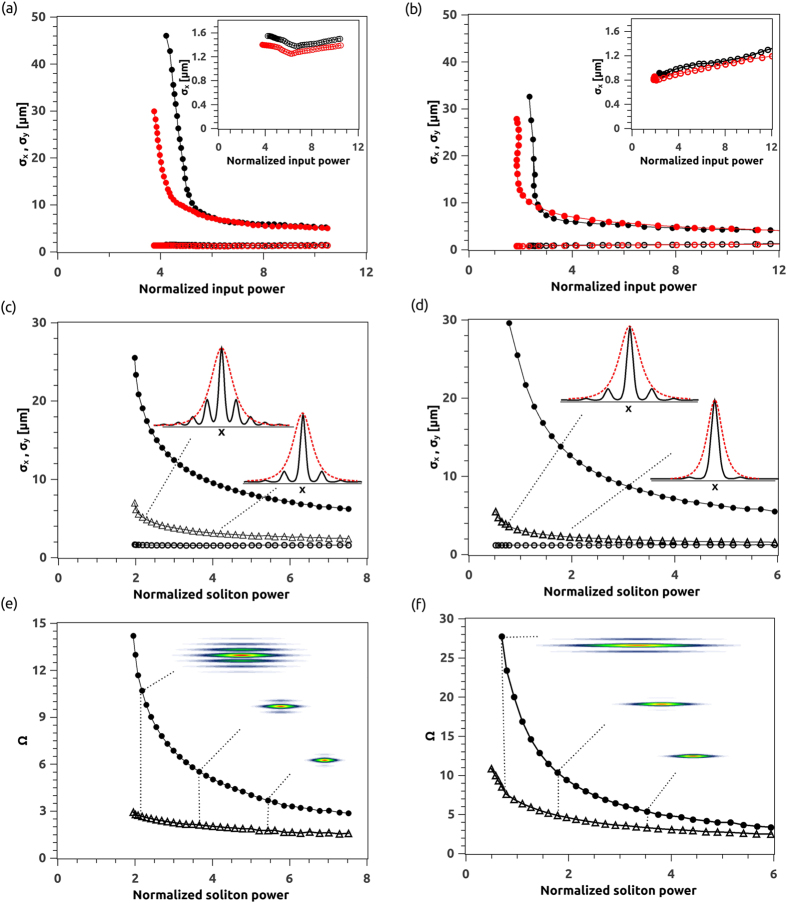
Beam Propagation Method: numerical results on astigmatic self-localized waves (a,c,e) in 1110 and (b,d,f) in 903. (**a**,**b**) Beam full-width half-maximum *σ* calculated in *z* = 2 *mm* along y (dots) and along x (circles) for an input beam with either *w*_0*x*_ = *w*_0*y*_ = 2.5 *μm* (black symbols, black lines) or *w*_0*x*_ = 3 *μm* and *w*_0*y*_ = 4 *μm* (red symbols, red lines), respectively. The insets show a magnification of *σ*_*x*_, with slight changes due to the nonlinear deformation of the guiding layer. (**c**,**d**) Beam full-width half-maximum *σ* versus soliton power across *y* (dots) and across *x* accounting for either the discrete beam envelope (triangles) or the mode of the excited (input) waveguide only (circles), respectively. (**e**,**f**) Orthogonal astigmatism of the normal modes in (**c**–**d**) versus soliton power for the single waveguide (triangles) or the discrete envelope (dots). Here we used the normalized power previously defined in Fig. 4.

Finally, using the (imaginary time) beam propagation method and solving for the first nonlinear mode (a single-hump soliton), we studied the astigmatism of these quasi-2D self-localized beams by calculating the full-width at half maximum *σ* of the beam amplitude profile in each transverse coordinate and the resulting astigmatism after propagation along *z* for 2 *mm*. The results are summarized in Fig. 7 for both ChNLC mixtures 1110 and 903, respectively. Figure 7(a,b) shows the computed FWHM across *x* (*σ*_*x*_, circles) and *y* (*σ*_*y*_, dots) for a nonlinear beam launched in the circularly symmetric case with *w*_0*x*_ = *w*_0*y*_ = 2.5 *μm* (black symbols) and in the elliptical case with *w*_0*x*_ = 3 *μm*, *w*_0*y*_ = 4 *μm* (red symbols), respectively. Clearly, the waist in the discrete array across *x* varies much less than across *y* due to the presence of the discrete structure, with minor variations due to the nonlinear deformation of the potential well versus power. Figure 7(c,d) graphs the FWHM of the localized normal mode across y (black dots) and *x* considering either the envelope of the discrete beam (more than one layer in the array, triangles) or the mode width in just the excited waveguide (circles). The latter remains substantially constant versus soliton power, as expected for the one-layer discrete soliton of the waveguide array. Figure 7(e–f) illustrates the asymptotic evolution of the (simple, orthogonal) beam astigmatism (Ω = *σ*_*y*_ /*σ*_*x*_ − 1) as computed from Fig. 7(c–d) 32, calculating *σ*_*x*_ from the discrete field envelope (dots) or the field guided in the input layer (triangles). As expected, the astigmatism of the nonlinear localized mode tends asymptotically to a finite value, which is comparable in both soft materials due to dual diffraction in the structure.

## Conclusions

In conclusion, we experimentally investigated light localization in a novel soft-matter chiral metastructure encompassing dual diffraction, i.e. continuous one-dimensional diffraction in one transverse dimension and quasi 1D discrete diffraction in the orthogonal direction. A self-focusing response enables confinement in both directions, giving rise to a quasi two-dimensional self-trapped beam with some degree of astigmatism, the latter stemming from dual diffraction combined with the distinct roles of the nonlinearity in the two dimensions. The results, obtained in a couple of specific molecular material systems exhibiting a reorientational response, namely chiral nematic liquid crystals, confirm the formation of astigmatic spatial solitons and are in excellent agreement with numerical simulations from a simple model featuring all-optical molecular reorientation, nonlocality and birefringence. The astigmatism of these quasi-2D solitons can be adjusted based on the form factor of the input excitation as well as on the birefringence of the medium.

## Methods

### Sample and experimental setup

In the experiments we used a linearly-polarized Gaussian (*TEM*_00_) beam from a Ti:Sapphire laser operating at *λ* = 793 *nm*. The extraordinary electric field was polarized along *y* and the beam was gently focused with a 20 x microscope objective to a waist of several micrometers at the input (*z* = 0) of the planar sample. Another microscope objective was placed at the cell exit and a charge-coupled-device (CCD) camera collected the beam profile in the plane *xy* at the output; a linear polarizer in front of the CCD allowed checking the polarization state after propagation. A second CCD camera was used to monitor the beam evolution along *z* by imaging the light out-scattered from the observation plane *yz*. The input beam position and profile were monitored by acquiring the reflected image with a third CCD camera. The planar cell was realized with 1.1 *mm* thick BK7 glass slides; the propagation length was 3 mm long along *z*. The inner glass interfaces were spin-coated with a 100 nm-thick polyimide alignment layer and then unidirectionally rubbed along *y* with soft fabric. The rubbing process caused local heating and stretching of the polyimide layer, in turn aligning its main chains and forming microgrooves for the preferential layout of the NLC molecules in the same direction. The liquid crystal molecules, in fact, tend to lie parallel to the rubbing direction in order to minimize the surface energy. The two glass plates were glued together with UV curable glue and spherical spacers to ensure a thickness of 40 *μm* across *x*. The cells were then filled up with ChNLC mixtures by capillarity and examined under a polarization microscope to check the proper alignment of the director as well as its homogeneous distribution.

We employed low- and high-birefringence nematic liquid crystals, respectively, synthesized *ad hoc* by R. Dabrowski and collaborators^33^. In the former case, we specifically used the NLC mixture 1110 with *n*_⊥_ = 1.45 and *n*_||_ = 1.49 at room temperature and wavelength *λ* = 793 *nm*; the Frank elastic constants in 1110 are *K*_11_ = 19.2 *pN*, *K*_22_ = 8.4 *pN* and *K*_33_ = 20.8 *pN* for splay, twist and bend deformations, respectively, whereas the critical temperature (nematic/isotropic transition) is *T*_*c*_ = 45.4 °C. In the latter case we employed the NLC mixture 903, exhibiting *n*_⊥_ = 1.47 and *n*_||_ = 1.55 at room temperature and *λ* = 793 *nm*; the Frank elastic constants in 903 are *K*_11_ = 16.1 *pN*, *K*_22_ = 7.3 *pN* and *K*_33_ = 17.7 *pN* for splay, twist and bend, respectively, and its critical temperature *T*_*c*_ = 65.9 °C. The same chiral dopant (5.43%) in both NLC mixtures resulted in a pitch *p* = 8 *μm*; the latter was measured using a standard Gradjean Cano technique in wedged cells^34^.

## Additional Information

**How to cite this article**: Laudyn, U. A. *et al.* Quasi two-dimensional astigmatic solitons in soft chiral metastructures. *Sci. Rep.* **6**, 22923; doi: 10.1038/srep22923 (2016).

## Supplementary Material

Supplementary Information
