# Supplementary material for: Quasi two-dimensional astigmatic solitons in soft chiral metastructures
Source: Sci Rep. 2016 Mar 15;6:22923. doi: 10.1038/srep22923 (PMC4791636; doi:10.1038/srep22923)
Supplement: Supplementary Information [file srep22923-s1.pdf]

Supplementary material
Quasi two-dimensional astigmatic solitons in soft chiral metastructures

Urszula A. Laudyn[1], Paweł S. Jung[1], Mirosław A. Karpierz[1], and Gaetano Assanto[2,3]

[1]Warsaw University of Technology, Faculty of Physics, Koszykowa 75, PL-00662 Warsaw, Poland
[2]NooEL–Nonlinear Optics and OptoElectronics Lab, University “Roma Tre”, I-00146 Rome, Italy
[3]Optics Lab, Physics Department, Tampere University of Technology, FI-33101 Tampere, Finland

ABSTRACT

This document provides supplementary information to ”‘Quasi two-dimensional astigmatic solitons in soft chiral metastructures”’. It contains extra details on light-driven deformation of the periodic structure in chiral nematic liquid crystals.

Beam-induced metastructure deformation

When injecting a round Gaussian beam ($w_{0x} = w_{0y} = 2.5\ \mu m$) in the ChNLC metastructure in order to obtain a self-localized beam by compensating dual diffraction, we had to increase the input power up to 50 mW to achieve a one-dimensional spatial soliton in the plane yz (Fig. 1(a)). However, for such value of the excitation we observed that several graded-index waveguides in the chiral array got excited, i.e. the discrete nematicon already obtained at powers close to 30 mW destabilized. The discrete pattern observed across x, conversely, appeared asymmetrically displaced with respect to the input ChNLC layer. Otherwise stated, as the input powers increased, dual diffraction was followed by discrete localization across x, then self-focusing across y, eventually 1D localization across y (Fig. 1(a)) and finally asymmetric coupling among guides (discrete diffraction) across x and overall aberration of the beam (Fig. 1(b)). Such a sequence of events is in contrast with the expected physical behaviour, whereby beam localization in the two transverse coordinates of the metastructure should lead to a bell-shaped quasi 2D self-confinement, as reported in the main text.

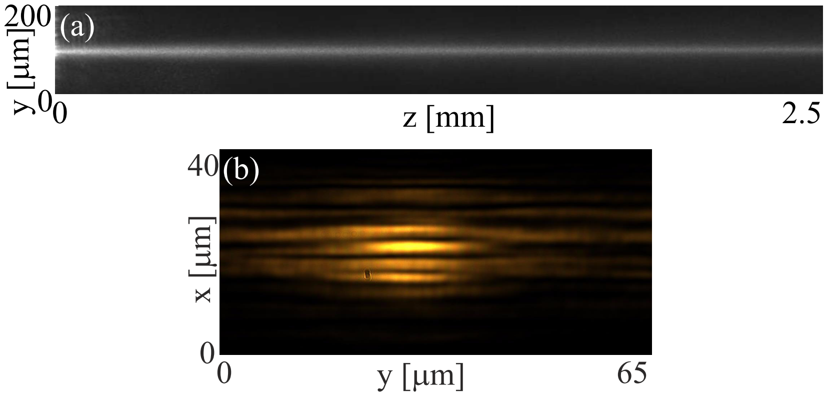

Figure 1. (a) Beam evolution in the plane yz for a 50 mW input beam in the chiral mixture 1110; (b) corresponding output (xy) profile in $z = 3\ mm$.

Aiming to understand the reasons of such unexpected trend versus input power, we carried out a set of observations with the aid of a polarizing microscope, imaging the layers of the ChNLC 1110 with white light as the input beam power was increased. As visible in (Fig. 2(a)), as the beam excitation reached 50 mW the periodic structure across x underwent an asymmetric deformation due to high power density, thereby causing nonuniform light coupling from the input guide to the adjacent waveguides in the region where their spacing was lowered by the distortion. The initially periodic structure defining the waveguide array across x became no longer periodic at high beam powers, with a net decrease in waveguide separation and coupling distance, as apparent from Fig. 2(b). Moreover, at high power beam the index contrast between waveguides as well

as their widths reduced through nonlocality and saturation of the reorientational response, in turn contributing to the overall destabilization of the discrete soliton generated at lower excitations.

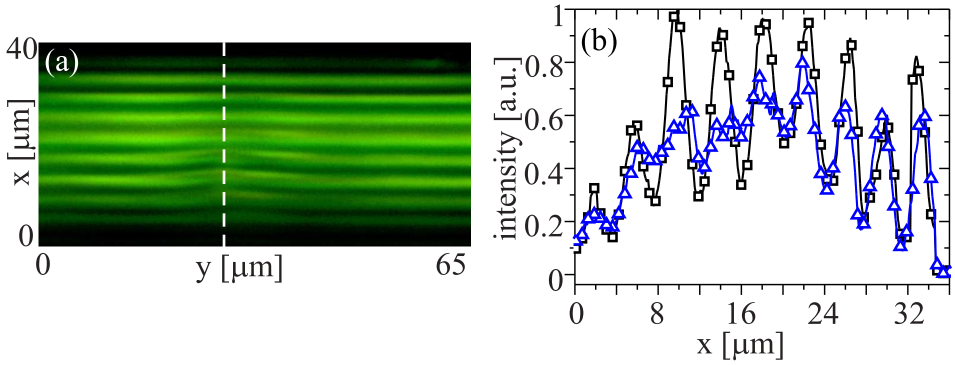

Figure 2. (a) Photograph of the asymmetrically deformed metastructure under white light illumination when excited by the beam (filtered out) in Fig. (1) ; (b) x cross-section of the metastructure (a) in $z = 3$ *mm* for various beam powers: black (squares) 1 mW, blue (triangles) 50 mW. The dashed line in (a) indicates the cross-section location.
